# Psychometric Properties of the Spanish Version of the Swallowing Quality of Life Questionnaire in Fibromyalgia Patients

**DOI:** 10.3390/healthcare13222948

**Published:** 2025-11-17

**Authors:** Irene Calles-Plata, Araceli Ortiz-Rubio, Laura Pérez-Gisbert, Irene Torres-Sánchez, Andrés Calvache Mateo, Marie Carmen Valenza, Alejandro Heredia-Ciuró

**Affiliations:** Department of Physiotherapy, Faculty of Health Sciences, University of Granada, Av. de la Ilustración 60, 18016 Granada, Spain; irenecalles11@correo.ugr.es (I.C.-P.); lauraperezg@ugr.es (L.P.-G.); irenetorres@ugr.es (I.T.-S.); andrescalvache@ugr.es (A.C.M.); cvalenza@ugr.es (M.C.V.); ahc@ugr.es (A.H.-C.)

**Keywords:** fibromyalgia, quality of life, psychometric properties, questionnaire, swallowing, validity

## Abstract

**Background and Purpose**: The Swallowing Quality of Life Questionnaire (SWAL-QoL) is recognized as the gold-standard instrument for assessing the impact of dysphagia on health-related quality of life (HRQoL). However, a validated Spanish version is currently unavailable. Therefore, the aim of this study was to evaluate the psychometric properties of the Spanish adaptation of the SWAL-QoL (SSWAL-QoL) in fibromyalgia patients. **Materials and Methods**: A cross-sectional study was completed. Participants completed the SSWAL-QoL, the Spanish version of Hospital Anxiety and Depression Scale (HADS), the Spanish version of Short Form12 (SF12), the Spanish version of EQ-5D-3L, and the Spanish version of the Impact Fibromyalgia Questionnaire (FIQ). Construct validity, evaluated through Exploratory Factor Analysis and correlations with related measures, and reliability, estimated via internal consistency, were found to be adequate for the SSWAL-QoL. **Results**: The results indicated that the psychometric properties of the SSWAL-QoL are adequate and comparable to those of the original instrument. The result of Bartlett’s test of sphericity was 970.573 (df = 55, *p* < 0.001), and the value of the Kaiser–Meyer–Olkin (KMO) measure of sampling adequacy was 0.857. A total of two factors could be extracted. The reliability was high for the total subscales of the SSWAL-QoL. Differences were found between groups in all included outcomes. **Conclusions**: The SSWAL-QoL demonstrates satisfactory psychometric properties for its use in a Spanish-speaking population with fibromyalgia. As the first validation of this instrument in this specific population, it provides clinicians and researchers with a reliable and valid tool to assess the impact of dysphagia on HRQoL.

## 1. Introduction

Fibromyalgia is a chronic condition characterized by widespread pain, musculoskeletal tenderness, fatigue, sleep disturbances, and emotional distress, affecting up to 8% of the global population [1]. While international diagnostic criteria remain varied in their nomenclatures and assessment method, the central pain component of the syndrome is a well-stablished contributor to reduced mental physical health, psychological state, perceived feelings about study or work, social life, well-being, and independence to function effectively [2]. This can result in job loss and, for some individuals, significant challenges in performing activities of daily living [3], including the essential tasks of maintaining adequate hydration and nutrition [4].

Although not formally recognized as a diagnostic criterion for fibromyalgia syndrome, self-reported difficulties with swallowing are emerging as a common subjective experience [5]. The last decades, a growing body of recent clinical studies has documented these individual complaints, suggesting that swallowing (medically term dysphagia) may be a prevalent yet under-recognized comorbidity in this population [6,7].

Despite these patient reports, the academic literature lacks a comprehensive characterization of swallowing difficulties in this population, including their clinical consequences, psychological impact, and optimal management strategies. A critical gap is the absence of a validated, fibromyalgia-specific instrument to assess dysphagia-related quality of life. Dysphagia, a condition marked by difficulty swallowing during the oral, pharyngeal, or esophageal stages, can lead to severe health complications such as dehydration, malnutrition, and aspiration pneumonia [8]. The drinking and eating difficulties reported by individuals with fibromyalgia include pain when swallowing, a sensation of food sticking (globus sensation), difficulty chewing, altered taste, oral burning sensation, and xerostomia [9].

The instruments designed for dysphagia in patients with head and neck cancer are the MADI [10,11]—validated against self-reported severity scores—and the DHI [12]. Each of these instruments differs in format and method of scoring. The most common tool used in research and clinical practice is SWAL-QoL [13]. While the SWAL-QoL remains the fold standard for evaluating the broader impact of dysphagia on HRQoL, adapted versions of these questionnaires exhibit divergent factor structures, even when their reliability is acceptable. The original SWAL-QoL questionnaire was developed by McHorney et al. [14] and revisited and validated by McHorney et al. in 2002 [15] to provide a patient-based, dysphagia-specific quality of life instrument for assessing patient variables and therapeutic efficacy. The original version showed outstanding psychometrics properties.

The SWAL-QoL [14,15] questionnaire is a seminal patient-reported outcome measure, comprising a total of forty-four items organized into eleven distinct subscales: general burden, eating desire, eating duration, symptoms, food selection, communication, fear of eating, mental health, social functioning, sleep, and fatigue. This robust structure ensures comprehensive coverage of all domain’s outlines by the World Health Organization’s International Classification of Functioning Disability and Health domains, making it a gold-standard instrument for evaluating the multifaceted impact of dysphagia on quality of life. Its widespread international adoption is evidenced by its rigorous cultural adaptation and validation into numerous languages, including Dutch [16,17], Chinese [18,19], French-Canadian [20], Swedish [21], Italian [22], Norway [23], Portuguese [24], German [25], and Greek [26]. Each of these versions underwent a formal process of adaptation and psychometric testing to ensure reliability, validity, and conceptual equivalence within their respective populations. Despite showing acceptable reliability, the factor structures of these adaptations differ. The psychometric evaluation of multiple translated SWAL-QoL versions confirms the scale’s status as a generally reliable and valid disease-specific HRQoL instrument. Internal consistency (α) is typically good to excellent, supporting group-level research (e.g., Mandarin Chinese α = 0.906; Greek α = 0.940). Test–retest reliability (ICC) demonstrates strong short-term stability across Swedish (ICC = 0.75–0.98) and Norwegian (ICC = 0.67–0.89).

However, a significant and limiting gap has persisted for Spanish-speaking clinicians and researchers. Although a Spanish-translated version existed years ago [27] and has been used for years in several populations, such as fibromyalgia [7], its psychometric properties have never been formally confirmed, and previous uses in Spanish-speaking chronic populations have relied on this unvalidated translation. This context underscores the unique, relevant, and novel contribution of this study to the literature. To our knowledge, even if there has been a growing interest in studying the effect of dysphagia on quality of life in chronic conditions, there is a remarkable lack of reliable instruments assessing repercussions of dysphagia on quality of life in the Spanish population. This is the need that this study sought to address: to evaluate the reliability and psychometric properties of the SWAL-QoL questionnaire among the Spanish population. The objective of this study was to explore and to report the psychometric properties of the Spanish version of the SWALQoL (SSWAL-QoL) in fibromyalgia patients.

## 2. Materials and Methods

### 2.1. Design

A cross-sectional study was completed. This study was conducted in accordance with the Declaration of Helsinki for experiments involving humans [28], and the protocol of this study was approved by the Biomedical Research Ethics Committee (1990/CEIH/2021).

### 2.2. Participants

The study population was composed of a series of individuals diagnosed with fibromyalgia, matched by age and sex with a control group. Patients with a neurological disorder, other rheumatic pathologies, or no understanding of the Spanish language were excluded. This study also included a sample of healthy volunteers (control group) recruited from circles of family and friends of the research group.

The inclusion criteria for the case group were the following: outpatients over 18 years old with a diagnosis of fibromyalgia [29] made by a rheumatologist. Eligibility for the control group stipulated that individuals were aged over 18 years, free from medical conditions affecting oropharyngeal motor or sensory performance, and without dysphagia, in accordance with previous recommendations [23]. For all prospective participants, exclusion criteria encompassed the presence of clinical conditions known to cause dysphagia (other than fibromyalgia in the patient group), cognitive impairment, a non-alert state, illiteracy in Spanish, or an unwillingness or inability to provide informed consent.

### 2.3. Data Collection

The participants with fibromyalgia completed the SSWAL-QOL, which was translated and culturally adapted into Spanish [27], the Spanish version of Hospital Anxiety and Depression Scale (HADS) [30,31], the Spanish version of Short Form12 (SF12) [32,33], and EQ-5D-3L [34,35,36]. We collected information on gender, age, education level completed, years with pain, and impact of fibromyalgia using the Spanish version of Impact Fibromyalgia questionnaire (FIQ) [37]. The control group completed all the instruments, except the instrument specific for individual with fibromyalgia, the FIQ. The control group completed the dysphagia-specific items primarily to (1) establish normative response patterns in control participants, which helps interpret patient scores and assess the discriminant validity of these measures; (2) maintain consistent administration procedures across all participants to avoid introducing procedural bias; and (3) enable direct statistical comparisons between groups on all measures.

### 2.4. Measures

FIQ [37]. The FIQ is a 10-item, self-administered questionnaire used to assess the impact of fibromyalgia on an individual’s life. It registers characteristics such as physical function, job difficulty, depression, anxiety, pain stiffness, fatigue, and overall well-being. The maximum score is 100, higher scores reflecting greater impact on the person. This questionnaire is available in Spanish and demonstrates adequate psychometric properties.

SSWAL-QoL [27]. The SWA-QoL was developed and validated by McHorney et al. in 2002 and cross-culturally adapted to Spanish patients. The authors employed a rigorous, multistage process to ensure linguistic and cultural equivalence. It began with independent dual forward translation by bilingual experts, which was synthesized into a single reconciled version. This version was then back-translated into the original language, English, by two additional blinded translators to identify discrepancies. A multidisciplinary expert committee meticulously reviewed all versions—original, forward and back translations, and the reconciled draft—to resolve any inconsistencies, achieving cross-cultural equivalence. The resulting preliminary questionnaire was pilot-tested with a dysphagia patient sample for comprehension and relevance. Final minor adjustments, informed by this patient feedback, yielded the official Spanish version, meticulously crafted for clarity and appropriateness for the target population. However, the authors did not explore the psychometric properties of this translated instrument. We used the Spanish version of the SWAL-QoL questionnaire. It consists, similar to the original version, of forty-four items that are categorized into the following eleven subscales: burden, food duration, eating desire, symptom frequency, food selection, communication, fear, mental health, social functioning, fatigue, and sleep. The Spanish-translated version was used in this study. The original scale showed that Cronbach’s alpha for the 11 domains ranged from about 0.79 to 0.94.

HADS [30,31]. HADS is a self-reported scale used to measure both anxiety and depression in patients with illness, as well as the general population. It consists of 14 items, divided into two subscales: HADS-A for anxiety and HADS-D for depression. Each item is scored on a 4-point Likert scale (0–3), with higher scores indicating greater severity. This questionnaire is available in Spanish and demonstrates adequate psychometric properties.

SF12 [32,33]. The SF-12 (Short Form-12 Health Survey) is a concise, self-reported instrument designed to measure health-related quality of life (HRQoL), serving as an efficient alternative to the longer SF-36. Comprising just 12 items, it assesses physical and mental health via two summary scores: the physical component summary (PCS) and the mental component summary (MCS). The PCS covers aspects such as physical functioning, bodily pain, and general health, while the MCS assesses vitality, social functioning, and emotional well-being. Scores are standardized, with a mean of 50 and a standard deviation of 10 in the general population. Psychometrically, the SF-12 demonstrates strong reliability (with Cronbach’s alpha typically exceeding 0.70) and validity, showing high correlations with the SF-36 and other HRQoL measures. Its brevity and robust psychometric properties make it a preferred tool in clinical research, population health studies, and cost-effectiveness analyses, where a balance between precision and efficiency is essential. This questionnaire is available in Spanish and demonstrates adequate psychometric properties.

EQ-5D-3L [34,35,36]. The EQ-5D-3L is an HRQoL instrument consisting of two parts: a descriptive system and a visual analogue scale (VAS). The descriptive system assesses five dimensions (mobility, self-care, usual activities, pain/discomfort, and anxiety/depression), each related on a three-level scale of severity (no problems, some problems, extreme problems). Patients indicate their health state by selecting the most appropriate level of each dimension. For these five dimensions and for the total score, a higher score indicates a greater level of problems or worse health status. The second component, the EQ-5D-3L-VAS, allows patients to self-rate their overall health on a vertical scale ranging from 0 “worst imaginable health state” to 100 (“Best imaginable health state”). The decision results into a 1-digit number that expresses the level selected for that dimension. This questionnaire is available in Spanish and demonstrates adequate psychometric properties.

### 2.5. Sample and Procedure

The Spanish version of SWAL-QoL was used in this study. This study adopted a cross-sectional, nonexperimental design. A convenience sampling method was used for participant recruitment from local associations and outpatient clinics. The recruitment process was conducted in collaboration with a consortium of local patients and community associations. A multimodal strategy was carried out to ensure broad outreach, encompassing three primary methods: (1) direct engagement through associated clinics and community centers, (2) dissemination of printed informational leaflets and flyers at relevant events and locations, and (3) digital advertisement via official announcements posted on the website and social media platforms of the partner associations.

Sample size was calculated according to the recommendation for conducting a robust Exploratory Factor Analysis (EFA). The literature suggests a minimum of 5 to 10 participants per questionnaire item [38,39]. The original version of the SWAL-QoL questionnaire comprises 44 items. Consequently, the 302 participants constitute a sample that complies with the standard recommendation.

### 2.6. Analysis

#### Statistical Analysis

Statistical analysis was carried out using IBM SPSS statistics for Windows, version 23 (IBM Corp., Armonk, NY, USA). An exploration of normality was performed using the Kolmogorov–Smirnov test. Bartlett’s test of sphericity was performed to evaluate whether the correlation matrix of the sample was a single identity, and whether it was suitable for factor analysis. Second, the value of the Kaiser–Meyer–Olkin (KMO) test was calculated with the sample of patients with fibromyalgia. Third, an EFA of principal-axis factoring was performed to explore the latent construct of the SSWAL-QoL. The procedure was applied to the scores on the 11 subscales among fibromyalgia participants. Eigenvalues greater than one and the scree plot were used to decide the number of salient factors in this study. Factor rotation was performed using promax rotation. This rotation was used because we theorized that the domains of the instrument are psychologically interrelated and should not be artificially constrained to be independent. To determine convergent and discriminant validity, a correlation analysis was developed. To establish a robust assessment of construct validity, the relationships between the SSWAL-QoL factors and validated external measures were examined a priori. The HADS, the SF-12, and the EQ-5D-3L were selected for their capacity to evaluate distinct yet related constructs, thereby enabling comprehensive testing of both convergent and discriminant validity. A specific pattern of relationships was hypothesized based on the theoretical definition of the SSWAL-QoL instrument. For convergent validity, we expected the SSWAL-QoL to demonstrate a strong negative relationship with both the HADS-anxiety and HADS-depression subscales. Concurrently, we predicted a strong positive relationship between the SSWAL-QoL and both components of the SF-12. Additionally, we anticipated that the SSWAL-QoL would show a strong negative relationship with the subscales of EQ-5D-3L and a positive relationship with EQ VAS. Correlations were considered strong with rho values ≥ 0.7, moderate with values between 0.3–0.7, and weak with values < 0.3 [33]. Internal consistency reliability for each instrument was evaluated using Cronbach’s alpha, applying a threshold of 0.70 for satisfactory reliability [40].

## 3. Results

### 3.1. Participant Characteristics

The sample was composed of 151 individuals with fibromyalgia (n = 142; 94% women) recruited through the associations, and a control group of 151 individuals (n = 142; 94% women). All participants completed the evaluation without receiving compensation. After being presented with the study terms, they provided informed consent and proceeded to respond to the measures. The demographic information for both groups is presented in Table 1.

### 3.2. Characteristics of the Score Distribution

The score distribution of the eleven scales of the instrument SSWAL-QoL are presented in Table 2. The analysis covered the full spectrum of possible scores (0–100) across all assessed scales. Distribution analyses indicated significant deviations from normality for all scales, as evidenced by skewness coefficients that markedly diverged from zero in both positive and negative directions.

This observation might be attributable to the clinical profile of the sample population and the characteristics of the sample population, which consisted of individuals with fibromyalgia presenting mild-to-moderate symptom severity.

### 3.3. Statistical Assessment of Construct Validity

An EFA was conducted to examine the underlying factor structure of the instrument. The suitability of the data for EFA was confirmed by a significant Bartlett’s test of sphericity 970.573 (df = 55, *p* < 0.001) and a Kaiser–Meyer–Olkin (KMO) measure of 0.856, exceeding the recommended threshold of 0.7. Using principal axis factoring for factor extraction, the analysis yielded two factors with eigenvalues greater than 1.00. These factors were rotated using an oblique rotation, acknowledging the potential for correlation between underlying constructs. The two-factor solution accounted for a cumulative 64.39% of the total variance.

As presented in Table 3, the pattern matrix revealed a simple and clinically interpretable structure. The first factor comprised the dysphagia-specific quality-of-life subscales, while the second factor included general measures, such as fatigue and sleep. The internal consistency for both factors was excellent, with Cronbach’s alpha values of 0.94 and 0.80, respectively. The factors demonstrated a low positive correlation (r = 0.30), suggesting that, while the dysphagia-specific quality-of-life factor and fatigue and sleep factor are related, they represent distinct constructs in this population.

### 3.4. Convergent and Discriminant Validity: Construct Validity

The construct validity of the instrument was assessed by examining its correlations with other instruments. Convergent validity was strongly demonstrated, as both factors showed significant correlations (*p* < 0.01) in hypothesized directions with related constructs. Specifically, Factor 1 (dysphagia-specific quality) and Factor 2 (fatigue and sleep) correlated negatively with HADS and EQ-5D-3L and positively with SF12 and EQ-5D-3L VAS. The systematically stronger correlations observed for Factor 2 across most criteria suggest it may tap into a broader health burden construct. While the provided data limits a full assessment of discriminant validity, the pattern of varying correlation magnitudes (e.g., stronger associations with physical health domains than with mental health) provides preliminary evidence that the instrument distinguishes between related but distinct constructs (see Table 4).

### 3.5. Internal Consistency

The internal consistency varies from 0.812 to 0.971; therefore, the instrument has general moderate–good reliability (see Table 5).

### 3.6. Clinical Validity

As hypothesized, the participants with fibromyalgia demonstrated significantly lower scores than the control group on the majority of SSWAL-QoL (*p* < 0.001), with the most pronounced difference observed in the symptom frequency subscale (see Table 6).

## 4. Discussion

The objective of this study was to explore and to report the psychometric properties of the Spanish version of the SSWALQoL in fibromyalgia patients. The findings confirm that the instrument evidences strong reliability and validity within this population, supporting its use in both clinical and research contexts in Sapin. The EFA revealed a two-factor structure, which collectively accounts for more than 64% of the variance in dysphagia-related quality of life. This model showed excellent internal consistency, as evidenced by high Cronbach’s alpha values (>0.70).

The two-factor structure identified in this study aligns with the conceptual dichotomy model observed in the original SWAL-QoL instrument validation [14,15] and is further supported by findings from Lai et al. in a Chinese Mandarin population with dysphagia [19], which also reported a two-factor structure. A key methodological commonality that explains this consistency is that both our study and the Chinese validation used the 11 subscale scores as input for the EFA. This approach, which operates at a higher level of abstraction, naturally consolidates the original subscales into a more parsimonious, higher-order factor structure. Moreover, the internal consistency estimated in our study was similar to that reported by Lai et al., reinforcing the cross-cultural reliability of the SSWAL-QoL’s score dimension in the Spanish population.

However, it is noteworthy that other cultural adaptations, such as the Dutch version [17], have yielded a different factor structure, with six factors explaining a substantial proportion of the variance. A critical methodological distinction likely underpins these divergent findings: the nature of the input data used for the Exploratory Factor Analysis (EFA). Our study and the Chinese [19] validation performed the EFA on the 11 subscale scores, effectively exploring the higher-order structure among the established domains. In contrast, the Dutch [17] study conducted the EFA on the raw data from all 44 individual items, which explores the underlying structure within the entire item pool. This is not a minor technicality but a fundamental difference in research questions. The latter approach (item-level EFA) is a more exploratory technique that seeks to discover how all individual questions naturally cluster, potentially revealing new, more granular domains that may not align perfectly with the original subscale design. Therefore, the six-factor solution found in the Dutch study represents a fine-grained, item-derived structure, while our two-factor solution represents a higher-order, domain-derived structure. Both are valid, yet answer different questions. These discrepancies might reflect cultural, linguistic, or methodological differences in adaptation and sample characteristics, highlighting the relevance of validating PROs within specific populations and cultural context [41].

In our study, it is relevant to elucidate that differences in smoking patterns between the individuals with fibromyalgia and the control participants were found. Specifically, the individuals with fibromyalgia reported higher rates of smoking compared to the control group. This information adds to the growing body of literature suggesting behavioral and lifestyle differences in fibromyalgia population, which may intersect with symptom burden and quality of life [42,43]. To our knowledge, other studies exploring the psychometric properties of SWAL-QoL do not report this information. Although the information was collected, the mechanisms underlying this association were not explored in the present study. It is plausible that smoking habits may serve as a coping mechanism for chronic pain or stress, or they may exacerbate certain symptoms such as xerostomia, potentially influencing dysphagia, perception, and quality of life [44,45]. Future research should further investigate the role of health behaviors, including smoking in dysphagia and quality of life in fibromyalgia, to better understand their clinical and prognostic implications.

To sum up, the Spanish version of the SWAL-QoL is a valid and reliable tool for assessing dysphagia-related quality of life in individuals with fibromyalgia. Its two-factor structure offers a clinically useful framework for evaluating all dimensions of swallowing difficulties. The instrument is well suited for use in Spanish-speaking clinical and research settings, where it may help monitor treatment outcomes and improve patient care. More studies are encouraged to confirm these findings in larger samples using confirmatory factor analysis.

## 5. Limitations

This study has certain limitations that should be mentioned. A primary limitation of this study is its reliance on convenience sampling, supplemented by snowball sampling, for participant recruitment. We explicitly and forcefully acknowledge that this approach severely limits the external validity and generalizability of our findings. Our sample was not randomly selected from the broader target population, but was instead drawn from readily accessible sources. We believe that the participants who self-selected into this study may systematically differ from the wider population of individuals with the fibromyalgia condition in critical ways, such as their level of motivation, engagement with healthcare services, or symptom severity. A second limitation concerning the methodological approach to our factor analysis should be mentioned: the number of factors were determined using the criterion of eigenvalues greater than one. However, more robust methods, such as Horn’s parallel test, should be applied in future analyses. A third limitation of this study is the use of a self-reported instrument. While self-report data is susceptible to biases such as social desirability and recall inaccuracy, we implemented several strategies to mitigate these concerns. For instance, we used a validated scale with proven reliability in the Spanish population, and we ensured participant anonymity to reduce social pressure. However, this study evidenced that patients with fibromyalgia report many symptoms of dysphagia, and their emotional and quality-of-life scores were considerably diminished compared with the control group.

## 6. Conclusions

In conclusion, this study is the first to explore psychometric properties and the underlying factor structure of the SSWAL-QoL questionnaire among Spanish-speaking individuals with fibromyalgia. The findings provide robust empirical evidence that the SSWAL-QoL is a reliable and valid instrument for assessing the subjective health-related quality of life in this clinical population. The SSWAL-QoL emerges as an essential tool for both clinicians and researchers in Spain, enabling the precise evaluation of swallowing-related quality of life and the ability to effectively track outcomes in response to therapeutic interventions for individuals with fibromyalgia.

## Figures and Tables

**Table 1:** Characteristics of participants included in this study.

Outcomes	Fibromyalgia Group (n = 151)	Control Group (n = 151)	*p* Value
Age (years)	53.94 (7.19)	51.81 (8.94)	*p* = 0.242
Sex (% women)	94%	94%	*p* = 1
Smoking (frequency)	94.0%	86.1%	0.042
Years with pain	19.01 (11.92)	-	-
Tender points actives	15.53 (3.72)	-	-
Impact of fibromyalgia (FIQ)	77.68 (16.48)	-	-
Education level			
- Basic - Secondary or equivalent - University or equivalent	39%	36%	
	26%	28%	
	25%	26%	*p* = 0.756

**Table 2:** Characteristics of the score distribution of subscales of SSWAL-QOL among individuals with fibromyalgia.

	Nº of Item	Range	Mean (SD)	Coefficient of Skewness	P25	P50	P75
Burden	2	0–100	76.33 (28.23)	−0.956	50.00	87.50	100.00
Food duration	2	0–100	68.91 (32.30)	−0.624	50.00	75.00	100.00
Eating desire	3	0–100	68.50 (30.20)	−0.725	41.67	75.00	100.00
Symptoms frequency	14	0–100	54.93 (18.51)	−0.760	42.65	60.29	70.59
Food selection	2	0–100	77.41 (32.14)	−1.218	50.00	100.00	100.00
Communication	2	0–100	78.00 (27.23)	−1.153	62.50	87.50	100.00
Fear	4	0–100	75.91(27.67)	−1.046	56.25	87.50	100.00
Mental health	5	0–100	84.10 (26.25)	−1.787	75.00	100.00	100.00
Social functioning	5	0–100	88.30 (24.48)	−2.364	90.00	100.00	100.00
Fatigue	3	0–100	31.11 (30.44)	0.874	6.25	25.00	50.00
Sleep	2	0–100	23.91 (31.26)	1.149	0.00	0.00	50.00

**Table 3:** Rotated factor loading for the SSWAL-QoL in the fibromyalgia sample.

	Promax Rotation	
Component/Factor	Factor 1 Dysphagia-Specific Quality	Factor 2 Fatigue and Sleep
Burden	0.932	
Food duration	0.833	
Eating desire	0.829	
Symptoms frequency	0.783	
Food selection	0.776	
Communication	0.683	
Fear	0.593	
Mental health	0.574	
Social functioning	0.478	
Fatigue		0.856
Sleep		0.748

Extraction method: principal axis factoring. Rotation method: promax with Kaiser normalization. Note: factor loadings < 0.40 are not presented. The correlation between factors was r = 0.30.

**Table 4:** Spearman correlation of the SSWAL-QOL with HADS, SF12, and EUROQ-5D-3L among individuals with fibromyalgia.

Instrument/Factor	Factor 1 Dysphagia-Specific Quality	Factor 2 Fatigue and Sleep
HADS-A	−0.619 **	−0.648 **
HADS-D	−0.675 **	−0.758 **
HADS-Total score	−0.695 **	−0.746 **
SF-12-physical	0.593 **	0.706 **
SF-12-Mental health	0.453 **	0.480 **
EQ-5D-3L-mobility	−0.577 **	−0.651 **
EQ-5D-3L-Personal care	−0.481 **	−0.494 **
EQ-5D-3L-Daily activities	−0.611 **	−0.685 **
EQ-5D-3L-pain	−0.621 **	−0.724 **
EQ-5D-3L-anxiety	−0.536 **	−0.629 **
EQ-5D-3L-Total score	−0.439 **	−0.486 **
EQ-5D-3L-VAS	0.419 **	0.455 **

** Correlation is significant at the 0.01 level (2-tailed).

**Table 5:** Internal consistency of the SSWAL-QOL.

SSWAL-QoL	Internal Consistency
Burden	0.925
Food duration	0.944
Eating desire	0.862
Symptoms frequency	0.915
Food selection	0.895
Communication	0.812
Fear	0.851
Mental health	0.968
Social functioning	0.971
Fatigue	0.920
Sleep	0.894

**Table 6:** Comparison between groups of SSWAL-QoL, emotional status and QoL.

Outcomes	Fibromyalgia Group	Control Group	*p* Value
SSWAL-QoL			
Burden	76.33 (28.23)	97.51 (7.50)	*p* < 0.001 *
Food duration	68.91 (32.30)	95.77 (12.15)	*p* < 0.001 *
Eating desire	68.50 (30.20)	95.19 (12.37)	*p* < 0.001 *
Symptoms frequency	54.93 (18.51)	80.65 (9.53)	*p* < 0.001 *
Food selection	77.41 (32.14)	99.50 (2.44)	*p* < 0.001 *
Communication	78.00 (27.23)	99.50 (2.44)	*p* < 0.001 *
Fear	75.91(27.67)	97.93 (6.91)	*p* < 0.001 *
Mental health	84.10 (26.25)	99.40 (3.55)	*p* < 0.001 *
Social functioning	88.30 (24.48)	99.90 (0.70)	*p* < 0.001 *
Fatigue	31.11 (30.44)	97.18 (8.27)	*p* < 0.001 *
Sleep	23.91 (31.26)	91.97 (18.94)	*p* < 0.001 *
Emotional status			
HADS-A	12.120 (4.08)	4.69 (3.24)	*p* < 0.001 *
HADS-D	9.680 (9.68)	1.91 (2.30)	*p* < 0.001 *
HADS–total score	21.800 (21.80)	6.62 (4.84)	*p* < 0.001 *
QoL			
SF-12-physical	27.828 (6.37)	52. (7.00)	*p* < 0.001 *
SF-12-mental health	38.177 (11.10)	53.253 (9.13)	*p* < 0.001 *
EQ-5D-3L-mobility	1.843 (0.46)	1.073 (0.26)	*p* < 0.001 *
EQ-5D-3L-personal care	1.510 (0.50)	1.000 (0)	*p* < 0.001 *
EQ-5D-3L-daily activities	1.961 (0.44)	1.073 (0.26)	*p* < 0.001 *
EQ-5D-3L-pain	2.490 (0.50)	1.351 (0.47)	*p* < 0.001 *
EQ-5D-3L-anxiety	2.059 (0.58)	1.132 (0.34)	*p* < 0.001 *
EQ-5D-3L-total score	21.735(12.23)	16.314 (13.99)	*p* < 0.001 *
EQ-5D-3L-VAS	36.373 (20.14)	68.272 (33.19)	*p* < 0.001 *

* Significant differences (*p* < 0.001).

## Data Availability

The original contributions presented in this study are included in the article. Further inquiries can be directed to the corresponding author.
